# Fifty Ologies That Concern the Dental Profession

**Published:** 1891-04

**Authors:** 


### EDITORIAL.

### Fifty Ologies that Concern the Dental Profession. Ology, Discourse, Treatise. A Science the Name of which Ends in Ology.

Adenology, a study of glands.

Aetiology, a study of the cause.

Angiology, a study of vessels of the body.

Arteriology, a study of arteries.

Anthropology, a study of the structure and functions of the body.

Arthrology, a study of joints.
Biology, a study of living things.
Bacteriology, a study of bacteria.
Chondrology, a study of cartilages.
Craniology, a study of structure and uses of skulls.
Dentology, a study of teeth.
Electrology, a study of electricity.
Embryology, a study of the embryo and its development.
Hæmatology, a study of blood.
Histology, a microscopical study of anatomy.
Homology, a study of similar parts.
Hydrology, a study of the properties and nature of water.
Hypnology, a study of sleep.
Iamatology, materia medica.
Lithology, a study of calcareous concretions.
Membranology, a study of membranes.
Micrology, a study of microscopical plants and animals.
Micristology, a study of the minutest organic fibres.
Morphology, a study of ideal forms of parts, or organs of plants and animals.
Neurology, a study of the nerves.
Nosology, a study of the classification of diseases.
Odontonosology, a study of the diseases of the teeth.
Organology, a study of the organs of the body.
Orismology, a study or explanation of technical terms of any science.
Osteology, a study of the bony system.
Otology, a study of the ear.
Phlebology, a study of the veins.
Physiology, a study of the laws of life and functions of living beings.
Pathology, a study of disease.
Psychology, a study of mental philosophy.
Pyretology, a study of fevers.
Semeiology, a study of the symptoms, or signs of disease.
Skeletology, a study of solid parts of body.
Somatology, a study of anatomy.

Spasmology, a study of convulsions.
Sphygmology, a study of the pulse.
Stomatology, a study of the mouth.
Symptomatology, a study of symptoms.
Syndesmology, a study of ligaments.
Technology, a study or explanation of terms and phrases belonging to the arts.
Teratology, a study of malformations and monsters.
Threpsology, a study of doctrine of nutrition.
Toxicology, a study of poisons.
Zoonosology, a study of diseases of animals.
Zymology, a study of fermentation. W.

